# Atypical Presentation of Sjogren’s Syndrome With Chronic Urticaria: A Case Report and Literature Review

**DOI:** 10.7759/cureus.35809

**Published:** 2023-03-06

**Authors:** Umair Ahmad, Tania Waseem, Kristin Logee

**Affiliations:** 1 Internal Medicine, Nuvance Health/Vassar Brothers Medical Center, Poughkeepsie, USA; 2 Internal Medicine, Nishtar Medical University, Multan, PAK; 3 Rheumatology, Nuvance Health, Poughkeepsie, USA

**Keywords:** inflammatory arthritis, hydroxycholoroquine, allergic disorder, chronic urticaria, sjogren’s syndrome

## Abstract

We present a case of a 26-year-old female with a history of 6-8 months of recurrent severe allergic reactions with anaphylaxis who presented with bilateral shoulder arthralgia and inflammatory arthritis of the right hand involving metacarpophalangeal joints (MCP). Physical examination was significant for tenderness in shoulder and knee joints, swelling, warmth, and tenderness involving MCP joints of the right hand. Schirmer’s test was positive in both eyes. Further workup revealed positive antinuclear antibody (ANA) (speckled pattern), anti-Ro antibodies, and elevated inflammatory markers including erythrocyte sedimentation rate (ESR) and C-reactive protein (CRP). The patient was diagnosed with Sjogren’s syndrome and was started on methotrexate once weekly and hydroxychloroquine with significant improvement in the symptoms. Also, we review the literature describing the association between chronic urticaria and rheumatological diseases. This case highlights the varied presentation of rheumatological diseases.

## Introduction

Primary Sjogren’s syndrome is an autoimmune disease that most commonly presents with exocrine glandular involvement with symptoms of keratoconjunctivitis and xerostomia resulting from lymphocytic infiltration and inflammatory changes in the lacrimal and salivary glands [1]. Approximately half of these patients have involvement of extra-glandular sites including joints, the nervous system, lungs, the gastrointestinal tract, and kidneys. The classification criteria for Sjogren’s syndrome have been published by the American and European rheumatological societies and include oral symptoms (at least one; dry mouth for at least three months, recurrent or persistently swollen salivary glands, and need for liquids to swallow dry foods), oral signs (at least one; unstimulated whole salivary flow ≤ 1.5 mL in 15 minutes, abnormal parotid sialography, and abnormal salivary scintigraphy), ocular symptoms (at least one; dry eyes for at least three months, foreign body sensation in the eyes, and use of artificial tears three or more times per day), ocular signs (at least one; abnormal Schirmer’s test without anesthesia and ≤5 mm/5 minutes, positive vital dye staining of the eye surface), histopathological features showing focal lymphocytic sialadenitis, abnormal sialography, and positive antibody tests (anti-Ro or anti-La or both) [2]. For the diagnosis, at least four of the six criteria should be present and must include either histopathological evidence or a positive antibody test. There have been several studies published that describe the association of chronic urticaria (more than six weeks) with autoimmune diseases. In this report, we present a case of Sjogren’s syndrome initially presenting as chronic urticaria and inflammatory arthritis.

## Case presentation

We present a case of a 26-year-old female with a history of 6-8 months of recurrent severe allergic reactions with anaphylaxis who presented with bilateral shoulder arthralgia and inflammatory arthritis of the right hand involving metacarpophalangeal joints (MCP). She was evaluated by an allergist and found to have elevated IgE levels with eosinophilia. She had been treated with antihistamines and completed multiple steroid tapers in the past without much benefit. Workup for celiac disease and inflammatory bowel disease was unrevealing.

**Figure 1:**
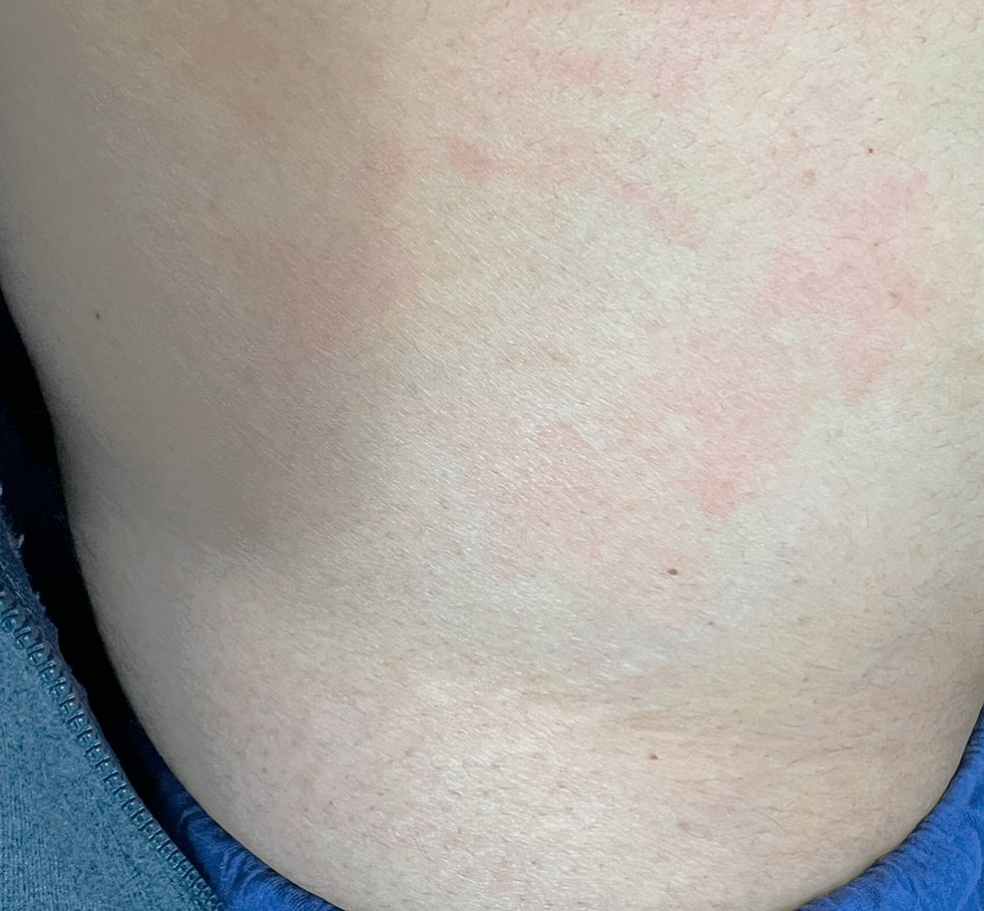
Urticaria on the abdominal area

At the time of presentation, physical examination findings were significant for tenderness in bilateral shoulder and knee joints, swelling, warmth, and tenderness in the MCP joints of the right hand with prominent involvement of the second and third metacarpophalangeal joints with the inability to make a fist. She had associated fatigue and decreased oral intake secondary to difficulty swallowing food attributable to dry mouth. The patient had patchy urticaria noted on the abdomen (Figure 1). Blood workup was significant for erythrocyte sedimentation rate (ESR) of 46 mm/hour (normal range: 0-29 mm/hour), C-reactive protein (CRP) of 2.1 mg/dL (normal range: <0.9 mg/dL), positive antinuclear antibody (ANA) with speckled pattern (1:160), and positive anti-Ro antibodies. The rest of the rheumatological workup was unremarkable. Radiological evaluation of the hands showed no joint pathology. For the symptoms of dry eyes, she was evaluated by the ophthalmologist and had a positive Schirmer’s test (left eye: 4 mm, right eye: 5 mm).

Based on the examination and blood workup, she was diagnosed with Sjogren’s syndrome. The patient was started on prednisone 10 mg daily along with once-weekly methotrexate, initially at a dose of 15 mg, which was increased to 25 mg, with moderate improvement in her symptoms. A few months later, the patient was started on hydroxychloroquine, significantly reducing the frequency of allergic reactions and arthralgia. The steroid therapy was tapered off over the course of a few months. For the symptoms of dry eyes and dry mouth, topical agents were prescribed, which were effective.

## Discussion

Sjogren’s syndrome is an autoimmune disease that commonly affects exocrine glandular structures, i.e., lacrimal and salivary glands, but it can also involve extra-glandular sites. Several studies have been conducted to describe the association between chronic urticaria and autoimmune diseases.

Chronic urticaria is characterized by the presence of urticaria (hives) for more than six weeks duration and occurs almost daily [3,4]. Literature review shows that around 50% of cases of chronic urticaria are associated with autoimmune diseases, resulting from the formation of autoantibodies to anti-IgE receptor alpha subunit (35%-40%) and anti-IgE (5%-10%) with increased frequency of human leukocyte antigen DR (HLA-DR) alleles [5]. This hypothesis was first proposed in 1986 when autologous intradermal injection resulted in wheal and flare reactions in some patients with chronic idiopathic urticaria [6]. Further studies showed that histamine-releasing activity was associated with autoantibodies to high-affinity IgE receptor alpha subunit, anti-IgE autoantibodies, or both [7].

A study conducted in Taiwan in 2010 showed that 10% of patients with chronic urticaria have a positive ANA, and subsequently, around 7.2% and 1.4% were found to have anti-Sjogren’s syndrome-related antigen A (SSA) and anti-Sjogren’s syndrome-related antigen B (SSB) antibodies, respectively. The study also highlights that the subset of patients diagnosed with rheumatic diseases had a higher prevalence of arthralgia [8]. In 2012, a large population-based study was conducted by Confino-Cohen et al. that showed that the most common autoimmune diseases associated with chronic urticaria were thyroid disease, followed by rheumatoid arthritis, in around 1.9% of female patients. The study also showed that the prevalence of Sjogren’s syndrome, celiac disease, systemic lupus erythematosus (SLE), and type 1 diabetes mellitus were significantly higher in female patients with chronic urticaria [9].

In the literature review, a few studies have been conducted that reported an increased frequency of allergic reactions in patients with Sjogren’s syndrome, especially to penicillin and sulfa drugs. A study conducted by Tishler et al. concluded that allergic reactions were more frequent in patients with Sjogren’s syndrome who have anti-Ro/SSA antibodies [10].

A study was published recently in 2022 that compared the efficacy of hydroxychloroquine and omalizumab in patients with chronic spontaneous urticaria, and the results were significant for complete response with hydroxychloroquine in two-thirds of the treated patients [11]. There has been a reported case of exercise-induced anaphylaxis that was successfully treated with hydroxychloroquine, which signifies the immunomodulatory effect of hydroxychloroquine and its use in autoimmune diseases [12].

Our case highlights that rheumatological diseases do not always present with the typical clinical features and require a more detailed physical examination and history taking to form a diagnosis. Early diagnosis is associated with improved outcomes and slowing of disease progression. In the past few decades, the understanding of autoimmune diseases has increased, leading to more advanced management options with disease-modifying agents that are proven to be more efficacious in controlling the disease progression long term.

## Conclusions

The case highlights the association of chronic urticaria and allergic reactions with Sjogren’s syndrome and emphasizes the importance of treating the underlying rheumatological disease in patients not improving with the conventional treatment for chronic urticaria. Rheumatological conditions can have varied presentations that usually lead to delayed diagnosis, but as a clinician, we should have high suspicion in cases where there is no improvement with conventional therapy. Early diagnosis of rheumatological diseases helps manage disease progression and improve quality of life.
